# Myxome géant de l'oreillette gauche appendue à la valve mitrale

**DOI:** 10.11604/pamj.2015.20.203.6333

**Published:** 2015-03-05

**Authors:** Achraf Zaimi, Nabil Berrada

**Affiliations:** 1 Service de Cardiologie, Hôpital Militaire Moulay Ismail, Meknès, Morocco

**Keywords:** Myxome, oreillette, valve mitrale, Myxoma, atrium, mitral valve

## Image en medicine

Le myxome cardiaque est la tumeur primitive intracardiaque la plus fréquente de l'adulte. Il est caractérisé par un polymorphisme clinique qui peut être déroutant pour le clinicien. L'avènement de l'échocardiographie en a bouleversé l'approche diagnostique. Malgré sa nature histologique bénigne, sa localisation intracardiaque engage le pronostic vital et impose une cure chirurgicale en urgence avec un risque de mortalité postopératoire minime. Le risque de récidive à long terme impose une résection large, une exploration minutieuse des quatre cavités et un suivi échocardiographique régulier. Nous rapportons le cas d'une jeune femme de 28 ans, sans aucun facteur de risque cardiovasculaire, reçue en consultation externe pour une dyspnée d'effort stade II de la NYHA évoluant depuis près de six mois. L'interrogatoire exclut une notion d'angines à répétitions dans l'enfance, de perte de connaissance et de déficit sensitivomoteur. L'examen physique révèle un roulement diastolique au foyer mitral. L'électrocardiogramme a montré un rythme sinusal, régulier à une fréquence cardiaque de 75 c/min avec une hypertrophie atriale gauche. Grâce à l'échocardiographie transthoracique, on a mis en évidence une masse insérée au niveau du septum interauriculaire occupant la totalité de l'oreillette gauche, hétérogène à surface irrégulière, appendue à la face atriale de la valve mitrale antérieure et traversant l'orifice mitral lors de la diastole et refoulée dans l'oreillette gauche pendant la systole. La patiente fut adressée directement à un service de chirurgie cardiovasculaire pour résection chirurgicale de la tumeur avec des suites simples.

**Figure 1:**
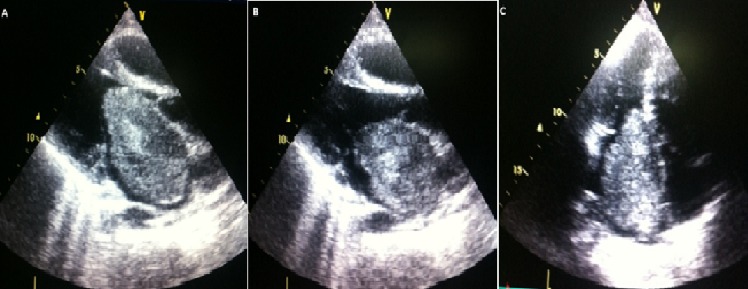
(A) myxome géant de l'OG lors de la diastole sur une coupe para sternale grand axe; (B) myxome de l'OG lors de la systole sur une coupe para sternale grand axe; (C) myxome de l'OG en vue apicale

